# Mechanical thrombectomy in stroke patients of working age: Real-world outcomes in Sweden

**DOI:** 10.1177/23969873211067883

**Published:** 2022-02-03

**Authors:** Mihae Roland, Ioanna Markaki, Tommy Andersson, Fabian Arnberg, Christina Sjöstrand

**Affiliations:** 1 Department of Clinical Neuroscience, 59562 Karolinska Institutet, Stockholm, Sweden; 2 Center of Neurology, Academic Specialist Center, Stockholm, Sweden; 3 Department of Medical Imaging, AZ Groeninge, Kortrijk, Belgium

**Keywords:** Thrombectomy, stroke, outcome, registry

## Abstract

**Introduction:**

Outcomes after mechanical thrombectomy (MT) in young stroke patients remain elusive due to small patient cohorts. We sought to determine outcomes after MT in stroke patients between ages 18 and 64 years and compare with outcomes in older patients in a large national stroke cohort.

**Patients and methods:**

We used the Swedish National Stroke Registry and the Swedish National Endovascular Thrombectomy Registry to identify all patients treated with MT for anterior circulation occlusions. We examined outcome measures in terms of functional independence at 90 days (modified Rankin Scale score of 0–2), symptomatic intracerebral hemorrhage (sICH), and mortality at 90 days with multivariable logistic regression analysis.

**Results:**

Of 2143 patients, 565 were between 18 and 64 years (26.4%) and 1179 (55.0%) were males. Analysis showed that patient aged 18–64 achieved higher rate of functional independence at 90 days (46.2% vs 28.4%, *p* < .001), had less often sICH (5.5% vs 6.8%, p = .008), and lower 90-day mortality rate (6.9% vs 17.7%, *p* < .001). Increasing age was associated with a lesser probability of functional independence at 90 days (adjusted odds ratio (aOR), 0.94; [95% confidence intervals (CIs) 0.93–0.95]), higher odds of mortality at 90 days (aOR, 1.05; [95% CIs 1.03–1.06]), and of sICH (aOR 1.03; [95% CIs 1.01–1.05]).

**Conclusion:**

Patients aged 18–64 years demonstrated better outcome after thrombectomy regarding functional independence, sICH, and mortality at 90 days when compared to older ages.

## Introduction

Over the last decades, reperfusion therapies (i.e., thrombolysis and/or thrombectomy) have proven successful for patients with acute ischemic stroke due to large vessel occlusion.^1^ In Sweden, the number of patients treated with mechanical thrombectomy (MT) has continuously increased every year. In 2019, 932 MTs were performed corresponding to about 5% of all ischemic stroke cases.^2^ Even though roughly a quarter of all patients that suffer from a stroke are under the age of 65 years, cohort studies on the outcome of MT in young stroke patients have been few and with a scarce number of patients.^3–5^ In addition to the individual suffering from many years with disability, stroke in young adults also lead to high costs for society in terms of rehabilitation and loss of work.^6^ This paper is part of an investigation aimed to map stroke in working ages (18–64 years) in Sweden. In this study, we sought to determine the rate of functional independence and mortality at 90 days as well as the risk for symptomatic intracerebral hemorrhage (sICH) in MT-treated patients <65 years of age compared with older age categories.

### Patients and methods

This is a national multi-center cohort study on patients treated with MT in Sweden between 2015 and 2019. The Swedish National Stroke Registry (Riksstroke) is prospectively maintained and comprises data on demographics, pre-stroke function, risk factors, and stroke care from primary- and comprehensive stroke centers in the entire country. The Swedish National Endovascular Thrombectomy Registry (EVAS) is a prospectively maintained endovascular registry for endovascular treatment of stroke with all six national MT-centers participating. Riksstroke and EVAS were in 2020 merged into the RS–EVAS database, forming an unselected national database for MT in Sweden from 2015 to 2019. Informed consent is not required for national quality registry-based studies. Registered patients are informed that they can opt-out from the registry.

The coverage for Riksstroke throughout the study years was 89–90% when controlled against the National Patient Registry.^2,7–10^ Mortality data in Riksstroke is collected through the Swedish Causes of Death Registry with >99% coverage.^2^ No coverage analysis was done for EVAS before 2018 as the national procedure code for MT was not gradually implemented nationwide until 2016. The coverage for EVAS for the years 2018 and 2019 were 96% and 98%, respectively.^11,12^ Data quality of the Riksstroke registry is ensured through the web-based registry platform as well as through statistical process control with manual evaluation of outliers.^13^ Validation studies have been made on different aspects of the Riksstroke registry.^14,15^ Validation of EVAS data is performed prior year report including cross-control of radiology and date- and time variables.^12^

Inclusion criteria for the study were all patients above the age of 18 registered in RS–EVAS with anterior circulation strokes (N = 2592). Patients with no remaining thrombus on digital subtraction angiography upon arrival at the MT center (N = 74), and patients with functional dependency (modified Rankin scale (mRS) > 2) prior to the stroke (N = 449) were excluded. In total, 2143 patients were included in the study. Hypertension was defined as ongoing treatment with anti-hypertensive medication upon admission. Smoking status included current smoking (at least one cigarette per day) or given up within the last 3 months. Diabetes (diabetes mellitus type 1 or 2) and atrial fibrillation (including intermittent or flutter) were either previously diagnosed or diagnosed before discharge.

### Outcome measures

Functional independence after stroke was measured at 90 days with the mRS. This score is computed by the Riksstroke registry, based on questions on dependency in daily activities in a questionnaire mailed to all patients three months after the stroke.^16^ Functional independence was defined as mRS 0–2. In addition, mortality at 90 days (mRS = 6) was extracted from Riksstroke data and presented as a separate outcome measure. Patients admitted from hospital to a care facility were classified as mRS >2. Successful recanalization was defined as modified Treatment In Cerebral Infarction score 2b–3. The incidence of sICH was assessed for each age group and defined as any hemorrhage detected by CT within 36 h accompanied with a worsening of the NIHSS score by four points or more.^17^

### Statistical analysis

We divided patients into two groups: 18–64 years old and ≥65 years old for comparisons of baseline characteristics and outcome. Continuous variables were assessed for normal distribution through the Kolmogorov–Smirnov test. Bivariate was analyses were performed using the Mann–Whitney U-test for ordinal variables, Student’s t-test for normally distributed continuous variables, and Fisher’s exact test for categorical variables. The outcome variable mRS at 90 days was dichotomized in functional independence (score 0–2) and dependence (score >2). Statistical significance was defined by two-sided values of *P* < .05.

We investigated the association between increasing age and outcome by performing logistic regression analyses on each outcome variable (mRS 0–2 and mortality at 90 days plus the presence of sICH) with age as continuous variable. Results from the logistic regression analyses are presented as odds ratios (ORs) with 95% confidence intervals (CIs). The linearity of each numerical explanatory variable on the outcome was controlled through the Box-Tidwell test and through graphical examination. The following variables were tested with univariate logistic regression against each outcome variable: age, sex, previous stroke, previous transient ischemic attack (TIA), hypertension, diabetes mellitus, atrial fibrillation, smoking, pre-procedure National Institutes of Health Stroke Scale (NIHSS), intravenous thrombolysis (IVT), onset to groin puncture, treatment duration, and successful recanalization. Significant variables from univariate regression were introduced to the multivariable model with the backward selection method and the results presented as adjusted odds ratios (aORs) with 95% CIs.

For further analysis we performed logistic regression analyses on each outcome variable with age as categorical variable. In addition to our cutoff at working age (<65), we use the definition of stroke in young adults (<50 years) and octogenarians (>80 years) in accordance with previous studies as additional cutoff points.^18–24^ Age was divided into the following subgroups: 18–49, 50–64, 65–80, and 81 and above. As ages 65–80 constitutes the main stroke incidence ages, this group served as reference group in comparisons with the other age groups with results presented in OR with 95% CIs.

Analyses were performed in SPSS version 26 (IBM, Armonk, New York) and RStudio version 1.3.1093 (RStudio, PBC 2020).

## Data availability

Anonymized study data can be made available upon request from a qualified researcher.

## Results

Of all patients (N = 2143; median age 73 years; IQR 64–80), 565 (26.4%) were between ages 18–64 and 1179 (55.0 %) were male. Baseline characteristics of the two age groups are presented in Table 1. Patients aged 18–64 reached functional independence at 90 days to a higher extent (46.2% vs 28.4%, *p* < .001). Furthermore, the 90-day mortality rate (6.9% vs 17.7%, *p* < .001) and the presence of sICH were lower in patients aged 18–64 years (5.5% vs 6.8%, *p* = .008). No significant differences were observed between the age groups for MT procedural time metrics, treatment with IVT, median change in NIHSS value at 24 h post-MT, or for the rate of successful recanalization. All vascular risk factors were less frequent in younger patients.

**Table 1.** Patient characteristics.

Variable	Overall sample *N* = 2143	<65 years (*n* = 565)	≥65 years (*n* = 1578)	*P* value
**Demographic characteristics**				
Median age (IQR)	73 (64–80)	56 (48–61)	77 (71–82)	
Male sex (No. (%))	1179 (55.0)	352 (62.3)	827 (52.4)	<.001
Single household (No. (%))	592 (27.6)	124 (21.9)	468 (29.7)	<.001
**Vascular risk factors (No. (%))**				
Hypertension	1237 (57.7)	194 (34.3)	1043 (66.1)	<.001
Atrial fibrillation	939 (43.8)	127 (22.5)	812 (51.5)	<.001
Diabetes mellitus	344 (16.1)	58 (10.3)	286 (18.1)	<.001
Smoking	288 (13.4)	138 (24.4)	150 (9.5)	<.001
Previous stroke	245 (11.4)	35 (6.2)	210 (13.3)	<.001
Previous TIA	140 (6.5)	18 (3.2)	122 (7.7)	<.001
**Medication (No. (%))**				
Platelet inhibitors	509 (23.8)	73 (12.9)	436 (27.6)	<.001
NOAC	192 (9.0)	38 (6.7)	154 (9.8)	.032
Wafarin	175 (8.2)	24 (4.2)	151 (9.6)	<.001
**Clinical characteristics**				
Pre-procedure NIHSS (median (IQR))	15 (10–19)	14 (9–18)	16 (11–20)	<.001
Level of consciousness (No. (%))				
alert	1585 (74.0)	448 (79.3)	1137 (72.1)	.001
drowsy	475 (22.2)	103 (18.2)	372 (23.6)	.001
comatose	45 (2.1)	7 (1.2)	38 (2.4)	.123
**Site of occlusion (No. (%))**				
Internal carotid artery (intra- and extracranial)	128 (6.0)	30 (5.3)	98 (6.2)	.471
Siphon, ophthalmic	29 (1.4)	6 (1.1)	23 (1.5)	.671
T-occlusion	288 (13.4)	77 (13.6)	211 (13.4)	.886
M1	1010 (47.1)	273 (48.3)	737 (46.7)	.523
M2, and more distally	602 (28.1)	157 (27.8)	445 (28.2)	.870
Anterior cerebral artery	86 (4.0)	22 (3.9)	64 (4.1)	1.000
**IVT**				
IVT (No. (%))	1139 (53.1)	313 (55.4)	826 (52.3)	.220
Onset to IVT in hours (median (IQR))	1.63 (1.25–2.25)	1.66 (1.18–2.18)	1.63 (1.27–2.25)	.222
Mothership (No. (% of IVT))	394 (34.6)	105 (33.5)	289 (35.0)	.676
Drip and ship (No. (% of IVT))	732 (64.3)	204 (65.2)	528 (63.9)	.729
**MT**				
Onset to groin puncture in hours (median (IQR))	3.45 (2.50–4.83)	3.26 (2.39–4.78)	3.52 (2.53–4.83)	.063
Treatment duration in minutes (median (IQR))	44 (27–74)	45 (27–72)	44 (26–74)	.680
Successful reperfusion (No. (%))	1763 (82.3)	459 (81.2)	1304 (82.6)	.091
**Outcome**				
sICH (<36 h) (No. (%))	138 (6.4)	31 (5.5)	107 (6.8)	.008
NIHSS median change	–6	–6	–6	.529
Mortality rate at 90 days all causes (No. (%))	319 (14.9)	39 (6.9)	280 (17.7)	<.001
Functional independence at 90 days^a^ (mRS 0–2) (No. (%))	709 (33.1)	261 (46.2)	448 (28.4)	<.001

Abbreviations: MT = mechanical thrombectomy; IVT = intravenous thrombolysis; mRS = modified Rankin Scale; NOAC = non-vitamin K antagonist oral anticoagulants; NIHSS = National Institutes of Health Stroke Scale; sICH = symptomatic intracerebral hemorrhage.

^a^Patients with pre-event disability (mRS >2) were excluded from the study material.

### Outcome analysis with age as continuous variable

#### Functional independence at 90 days

Of the 2143 patients, 335 (15.6%) were lost to follow-up at three months and were excluded from the analysis. Age distribution for missing patients were (mean (SD)): 66.7 (13.9) years. A total of 1808 patients (84.4%) were included in the univariate analysis and 709 (33.1%) patients reached functional independence. Increasing age was associated with lower odds for functional independence at 90 days (OR 0.94, 95% CI 0.93–0.95; Supplemental Table 1 in supplemental material), and (I) age (aOR 0.94, 95% CI 0.93–0.95), (II) male sex (aOR 1.47, 95% CI 1.15–1.87), (III) diabetes (aOR 0.35, 95% CI 0.24–0.50), (IV) pre-procedure NIHSS score (aOR 0.91, 95% CI 0.89–0.93), (V) IVT (aOR 1.58, 95% CI 1.24–2.01), (VI) treatment duration (aOR 0.99, 95% CI 0.98–0.99), and (VII) successful recanalization (aOR 3.29, 95% CI 2.21–5.00) were significant predictors of functional independence in the multivariable model (n = 1468; 68.5%).

#### Symptomatic intracerebral hemorrhage (sICH)

A total of 138 patients (6.4%) suffered from sICH. Information on sICH was missing in 327 (15.3%) patients including 55 patients with no assessment of NIHSS score at 24 h due to comatose state and 16 patients who died within 24 h of treatment.

All patients with available information on sICH, n = 1816 (84.7%), were included in the univariate analysis where age was associated with an annual increase of 2% in the OR for sICH (Supplemental Table 2 in supplemental material). The majority of patients (n = 79 (57.2%)) with sICH did not receive IVT. Patients with sICH had a median of 10 points on NIHSS at admission versus 15 points in patients without sICH. In the multivariable analysis (n = 1571; 73.3%), diabetes remained the strongest predictor for sICH (aOR 1.84, 95% CI 1.08–3.04), together with increasing age (aOR 1.03, 95% CI 1.01–1.05) and treatment duration (aOR 1.02, 95% CI 1.01–1.02). Increasing NIHSS score at admission (aOR 0.88, 95% CI 0.84–0.91), successful recanalization (aOR 0.29, 95% CI 0.18–0.47) and treatment with IVT (aOR 0.62, 95% CI 0.40–0.96) were correlated to lower odds for sICH.

#### Mortality at 90 days

In the univariate analysis all patients were included (Supplemental Table 3 in supplemental material). Age was associated with increased mortality at 90 days with a 6% increase in OR per year of age. From the univariate model, (I) age (aOR 1.05, 95% CI 1.03–1.06), (II) previous TIA (aOR 1.95, 95% CI 1.14–3.25), (III) diabetes (aOR 1.99, 95% CI 1.38–2.84), (IV) increasing pre-procedure NIHSS score (aOR 1.09, 95% CI 1.06–1.13), (V) IVT (aOR 0.54, 95% CI 0.42–0.79), (VI) successful recanalization (aOR 0.40, 95% CI 0.28–0.58), and (VII) sICH (aOR 3.95, 95% CI 2.44–6.36) remained significant in the multivariable model (1785 patients; 83.3%). Time metrics in the form of symptom onset to groin puncture and total treatment time were not associated with increased odds for mortality at 90 days. Patients with sICH had a four times increased likelihood for mortality at 90 days. IVT before MT reduced the odds for mortality by approximately 50%.

### Differences in outcome between age groups

For further comparisons, the cohort was divided into smaller age groups. Patients aged 18–49, 50–64, and 81 and above were analyzed through univariate logistical regression and compared to the age group 65–80 as a reference group as it comprises the most common stroke incidence ages; Table 2.

**Table 2.** Outcome analysis with age as categorical variable. Comparisons calculated with binary logistical regression analysis with age as categorical variable.

		Functional independence 90 days (mRS 0–2)		SICH (<36h)		Mortality 90 days
Ages, N	n	OR (95% CI)	N	OR (95% CI)	n	OR (95% CI)
65–80 (reference), *N* = 1083	938	–	915	–	1083	–
18–49, N = 65	48	3.90 (2.09–7.74)	56	0.48 (0.08–1.61)	65	0.29 (0.07–0.79)
50–64, N = 500	382	2.10 (1.65–2.68)	443	0.92 (0.57–1.43)	500	0.46 (0.31–0.67)
≥81, *N* = 495	440	0.25 (0.19–0.33)	402	1.53 (1.01–2.28)	495	2.02 (1.55–2.63)

Abbreviations: RS = modified Rankin Scale; sICH = symptomatic intracerebral hemorrhage.

When comparing treatment outcome to the reference group, all patients aged 18–64 have ORs favoring functional independence at 90 days and less probability of mortality at 90 days. Older patients (81 and above) carried a two times higher likelihood for mortality at 90 days than patients aged 65–80. No significant difference was found when comparing patients aged 18–64 to the reference group for sICH. Patients above 80 had a 1.5 times larger likelihood for sICH than the reference group.

## Discussion

In this national multi-center cohort study, we show that stroke patients aged 18–64 years (younger age group) demonstrate a significantly better outcome after MT in terms of functional independence and survival at 90 days when compared to older ages. As expected, all registered vascular risk factors for stroke were significantly fewer in the younger age group. However, other factors such as time metrics, occlusion location, successful recanalization, and IVT treatment including distribution modality (drip and ship vs mothership) were not different between the younger and older age groups.

Pooled analysis from five randomized trials (HERMES, N = 1278) showed a significant treatment effect for mRS at 90 days for all age strata except for patients 18–49 years (n = 158).^25^ We show that patients treated with MT aged 18–49 were four times more likely to achieve functional independence compared to ages 65–80 years. Moreover, a recent prospective cohort study (N = 264) on MT outcomes, with similar patient characteristics to our own, concluded that the outcome in real-world patient cohorts is likely to be generally worse than in the previously mentioned randomized control trial (RCTs) due to the much broader inclusion criteria.^26^ In line with this suggestion, our mortality rate across all ages (14.9%) was higher than some of the RCTs (9–18.9%).^27–31^ The odds for mortality in patients above 80 years was twice compared to patients aged 65–80. Similarly, inpatient mortality after MT for patients above 80 years has previously been determined twice their younger counterparts.^32^ In this study, we evaluate all-cause mortality which arguably should be most frequent in the oldest age strata regardless of MT outcome. Furthermore, the risk of sICH in our study was significantly higher for patients above the age of 80 years whereas the prevalence of sICH did not differ between ages 18–64 and 65–80 years. The reported SICH rate in this study is similar to previous pooled analysis,^33^ and the reported correlation of diabetes with sICH is in line with a previous systematic review.^34^

While relatively few studies have examined MT outcome in patients below 65 years of age, studies on patients above 80 years are more frequent. A meta-analysis of eight studies (N = 1711 patients) compared the outcome of patients dichotomized at 80 years of age.^20^ The OR for functional independence in patients above the age of 80 was 0.3, as compared to younger patients. Similarly, we present an OR 0.25 for functional independence in patients aged above 80 years with reference to patients aged 65–80 years. Moreover, a recent study (N = 169) showed that only 25.3% of patients above 80 years achieve functional independence after MT, with IVT a significant prognostic factor as in this study.^22^ Of note, two studies (N = 219, N = 80) suggest no significant difference in functional independence at 90 days when comparing patients above 80 years to younger ages whilst another study (N = 274) suggest age as a significant predictor of functional independence, which is in line with the results in our study.^21,23,24^

In the only three MT studies that were conducted on young stroke cohorts (ages 18–35 and 18–55) during the last decade, the results show remarkably high rates of successful recanalization and functional independence at 90 days, arguing that the treatment is particularly beneficial in this subset of patients.^3–5^ However, these studies have a low number of included patients (7, 15, and 45 patients, respectively) signaling limited statistical power and risking statistical type-I errors.

We note that IVT before MT was associated with better survival at 90 days. Bridging IVT was also associated with a higher rate of functional independence across all age strata. In patients ≥65 years, IVT was associated with less likelihood for sICH. As this material represents a real-world setting, the patients that received IVT before MT differ fundamentally from patients not eligible for IVT. One of the most common reasons for being unable to administer IVT, despite hospital arrival within 4.5 h of symptom onset, is ongoing prophylactic treatment with oral anticoagulants in patients with atrial fibrillation. Comparisons with recently published RCTs on bridging IVT before MT could be difficult due to this fundamental difference in the demographics of patients receiving IVT compared to those treated with direct MT.^35–37^ Moreover, this real-world material includes MT performed also in more distal locations in the vascular territory than those that are commonly the subject for RCTs.

A great strength of this real-world national cohort study is the large number of included patients which provide adequate statistical power for the evaluation of the selected outcome measures. Furthermore, this study includes all Swedish centers performing MT, thus comprising all patients treated during the investigation period and providing highly generalizable results. These results were based on data from two national quality registries with high coverage and extensive measures taken to ensure validity.

This study has several limitations. We used patient records that were not strictly designed for the purpose of the study, resulting in missing values especially regarding follow-up and it is possible that this loss might introduce bias in the results. Since the mean age for missing patients in the functional outcome analysis were lower than the cohort average, it is possible that there is an underestimation of the reported results. However, the demographics and clinical profile of our patients as well as the main results presented are in line with previously published large cohorts. Another limitation is that ASPECT scores were not included in the registries used, which introduces difficulties in comparing our results with other studies using exclusion criteria associated with ASPECT score. Lastly, we lack information about patients <18 years as they are not included in the registries used in this study.

## Conclusion

Good outcome after MT in terms of functional independence and survival at 90 days was strongly associated with ages 18–64, independently of other covariates. Consequently, the risk for a poor outcome increased significantly with age. Procedural time metrics, occlusion location, recanalization success, and treatment with IVT did not differ between the age groups.

## Supplemental Material

sj-pdf-1-eso-10.1177_23969873211067883 – Supplemental Material for Mechanical thrombectomy in stroke patients of working age: Real-world outcomes in Sweden Click here for additional data file. Supplemental Material, sj-pdf-1-eso-10.1177_23969873211067883 for Mechanical thrombectomy in stroke patients of working age: Real-world outcomes in Sweden by Mihae Roland, Ioanna Markaki, Tommy Andersson, Fabian Arnberg, and Christina Sjöstrand in European Stroke Journal

## Appendix

### Glossary

IVT: intravenous thrombolysis
mRS: modified Rankin Scale
MT: mechanical thrombectomy
NIHSS: National Institutes of Health Stroke Scale
sICH: symptomatic intracranial hemorrhage
