# Supplementary material for: Mechanical thrombectomy in stroke patients of working age: Real-world outcomes in Sweden
Source: Eur Stroke J. 2022 Feb 3;7(1):41–7. doi: 10.1177/23969873211067883 (PMC8921781; doi:10.1177/23969873211067883)
Supplement: sj-pdf-1-eso-10.1177_23969873211067883 – Supplemental Material for Mechanical thrombectomy in stroke patients of working age: Real-world outcomes in Sweden [file sj-pdf-1-eso-10.1177_23969873211067883.pdf]

Supplemental Tables – Supplemental material for Mechanical Thrombectomy in Stroke Patients of Working Age - Real-world Outcomes in Sweden

Description: Supplemental material for Mechanical Thrombectomy in Stroke Patients of Working Age - Real-world Outcomes in Sweden by Roland et al. in European Stroke Journal.

eTable 1. Univariate and multivariable regression modeling of patient variables for functional independence (mRS 0–2).

Variable	Univariate OR (95% CI)	Multivariable OR (95% CI)
Age	0.94 (0.93–0.95)	0.94 (0.93–0.95)
Sex (male)	1.42 (1.17–1.72)	1.47 (1.15–1.87)
Previous stroke	0.63 (0.46–0.86)	—
Previous TIA	0.72 (0.48–1.06)	—
Hypertension	0.49 (0.41–0.60)	—
Diabetes mellitus	0.35 (0.26–0.47)	0.35 (0.24–0.50)
Atrial fibrillation	0.55 (0.45–0.67)	—
Smoking	0.82 (0.61–1.10)	—
Pre-procedure NIHSS	0.91 (0.90–0.93)	0.91 (0.89–0.93)
IVT	1.74 (1.43–2.10)	1.58 (1.24–2.01)
Onset to groin puncture	0.93 (0.88–0.99)	—
Treatment duration	0.99 (0.98–0.99)	0.99 (0.98–0.99)
Successful recanalization	3.82 (2.80–5.31)	3.29 (2.21–5.00)

Abbreviations: IVT = Intravenous thrombolysis, mRS = modified Rankin Scale, NIHSS = National Institutes of Health Stroke Scale.

eTable 2. Univariate and multivariable regression modeling of patient variables for sICH.

Variable	Univariate OR (95% CI)	Multivariable OR (95% CI)
Age	1.02 (1.00–1.03)	1.03 (1.01–1.05)
Sex (male)	0.92 (0.65–1.30)	—
Previous stroke	1.56 (0.94–2.48)	—
Previous TIA	1.76 (0.94–3.06)	—
Hypertension	1.12 (0.79–1.61)	—
Diabetes mellitus	1.84 (1.20–2.75)	1.84 (1.08–3.04)
Atrial fibrillation	0.89 (0.62–1.27)	—
Smoking	1.02 (0.59–1.66)	—
Pre-procedure NIHSS	0.88 (0.86–0.91)	0.88 (0.84–0.91)
IVT	0.64 (0.45–0.91)	0.62 (0.40–0.96)
Onset to groin puncture	1.19 (1.07–1.32)	—
Treatment duration	1.02 (1.01–1.02)	1.02 (1.01–1.02)
Successful recanalization	0.23 (0.16–0.34)	0.29 (0.18–0.47)

Abbreviations: IVT = Intravenous thrombolysis, NIHSS = National Institutes of Health Stroke Scale, sICH = Symptomatic Intracerebral Hemorrhage.

eTable 3. Univariate and multivariable regression modeling of patient variables for mortality at 90 days.

Variable	Univariate OR (95% CI)	Multivariable OR (95% CI)
Age	1.06 (1.04–1.07)	1.05 (1.03–1.06)
Sex (male)	0.95 (0.75–1.21)	—
Previous stroke	1.52 (1.07–2.11)	—
Previous TIA	2.03 (1.34–3.01)	1.95 (1.14–3.25)
Hypertension	1.66 (1.29–2.15)	—
Diabetes mellitus	2.22 (1.67–2.93)	1.99 (1.38–2.84)
Atrial fibrillation	1.57 (1.23–1.99)	—
Smoking	0.87 (0.58–1.26)	—
Pre-procedure NIHSS	1.09 (1.07–1.11)	1.09 (1.06–1.13)
IVT	0.56 (0.44–0.72)	0.58 (0.42–0.79)
Onset to groin puncture	1.01 (0.94–1.09)	—
Treatment duration	1.01 (1.01–1.01)	—
Successful recanalization	0.35 (0.27–0.47)	0.40 (0.28–0.58)
sICH (<36h)	3.52 (2.33–5.23)	3.95 (2.44–6.36)

Abbreviations: IVT = Intravenous thrombolysis, mRS = modified Rankin Scale, NIHSS = National Institutes of Health Stroke Scale, sICH = Symptomatic Intracerebral Hemorrhage.
